# Effect of vitamin D on proteinuria in type 2 diabetic patients 

**DOI:** 10.15171/jnp.2017.03

**Published:** 2016-08-14

**Authors:** Ali Momeni, Mahmood Mirhosseini, Mohsen Kabiri, Soleiman Kheiri

**Affiliations:** ^1^Department of Internal Medicine, Shahrekord University of Medical Sciences, Shahrekord, Iran; ^2^Social Health Determinants Research Center, Shahrekord University of Medical Sciences, Shahrekord, Iran

**Keywords:** Vitamin D, Diabetes mellitus, Proteinuria, Diabetic nephropathy

## Abstract

**Background:**

Vitamin D (Vit D) deficiency is a common disorder in diabetic patients and may be a risk factor for ischemic heart disease and exacerbation of diabetic nephropathy(DN).

**Objectives:**

The aim of this study was to evaluate the effect of Vit D3 therapy on protein uriain type 2 diabetic patients with deficient or insufficient serum Vit D.

**Patients and Methods:**

In a double blind clinical trial, 60 type 2 diabetic patients with proteinuria greater than 150 mg/day who had Vit D deficiency or insufficiency were randomly enrolled in two equal groups. Pearl of Vit D as 50 000 IU/week and placebo (1 tablet per week) were prescribed in patients of case and control groups respectively for 8 weeks. At the beginning and 2 months later, 24 hours urine protein was checked in all patients.

**Results:**

There is no difference between serums Vit D level in case and control group at the beginning of the study, however at the end of the study serum Vit D level was significantly higher in the case group. There is no difference in proteinuria between case and control group at the beginning and the end of the study, while a significant difference between the changes of proteinuria before and after the study was seen in two groups (*P* = 0.028).

**Conclusions:**

Vit D deficiency may exacerbate protein uric and DN, hence correction of Vit D deficiency may decrease proteinuria in diabetic patients with nephropathy.

**Implication for health policy/practice/research/medical education:**

Nephropathy is a common complication of diabetes, so management of it could lead to decreasing of mortality and morbidity of the patients. In addition of angiotensin converting enzyme inhibitors and angiotensin receptor blockers, other drugs such as spironolactone, statins, and allopurinol were used for management of diabetic nephropathy with acceptable results in some studies. Vitamin D metabolites may have renoprotective and anti-proteinuric effect, decreasing of insulin resistance and blood pressure lowering effect, as well. Pearl of vitamin D is an inexpensive, safe and probably effective drug for patients with diabetic nephropathy and vitamin D deficiency.

## 1. Background

The prevalence of diabetes Mellitus (DM) worldwide was 2.8% and total number of patients was 171 million in 2000. There are continuing increase in the prevalence and incidence of DM, while the incidence of DM was 7.1% in 2012 (1). Diabetic nephropathy (DN) is the most common cause of chronic kidney disease and end stage renal disease, as about 30% to 35% of dialysis patients have diabetes (2). Diabetes is also the most common cause of renal replacement therapy requirement, in the United States (3). One of the common and serious complications of diabetes is nephropathy, defined by the development of proteinuria and classified based on the severity of proteinuria as microalbuminuria and macroalbuminuria. With onset of proteinuria, glomerular filtration rate decreased gradually at the rate of 10-12 mL annually or 1ml monthly (4,5). Lifestyle modifications (such as regular exercise, weight loss in obese patients, limitation of salt and alcohol intake, and restriction of dietary protein intake), blood pressure and serum glucose controlling may have a role in the prevention of DN (6). The basic drug treatment of DN is inhibition of the renin–angiotensin aldosterone system with ACE inhibitors or angiotensin II–receptor blockers. Combination of angiotensin converting inhibitors plus angiotensin receptor blockers (dual system blocking) was shown more effective than single agent therapy in some studies (7). There are a few novel modalities for treatment of DN, for example, aliskiren, a direct renin inhibitor has shown the anti-proteinuric effect in diabetic patients (8). Non-dihydropyridine calcium channel blockers such as diltiazem also have renoprotective effect probably due to decreasing hyperfiltration and intra-glomerular pressure (9), in addition, fenofibrate was shown to be effective in reducing proteinuria in small clinical trials (10). Serum level of uric acid may be greater in DN patients compared to normal population (11), thus allopurinol was shown to be effective in reducing proteinuria in type 2 diabetic patients (12). Spironolactone an aldosterone receptor blocker probably has renoprotective effect due to its anti-inflammatory property (13). In addition, combination of spironolactone and hydrochlorothiazide may be effective in DN treatment (14).

Vitamin D (Vit D) deficiency is a common disorder in diabetic patients and may be a risk factor for ischemic heart disease, deterioration of chronic kidney disease and DN (15).

Vit D metabolites may have a role in the inhibition of the renin-angiotensin system and renoprotective effect by preventing of glomerulosclerosis and anti-proteinuric effect. Furthermore, prescription of Vit D has shown decrease in insulin resistance and decrease in blood pressure as well (16,17).

## 2. Objectives

Recently, some studies were carried out regarding to effect of Vit D supplementation on reducing proteinuria in diabetic patients. However the results of these studies are controversial. Therefore, we aimed to evaluate effect of Vit D in reducing proteinuria in the type 2 diabetic patients with 25 (OH) Vit D deficiency or insufficient.

## 3. Patients and Methods

### 3.1. Study patients

In this double-blind randomized clinical trial, 60 type 2 diabetic patients were randomly enrolled in two equal case and control groups. Inclusion criteria were: proteinuria greater than 150 mg/dl, glomerular filtration ratio greater than 50 mL/min or serum creatinine < 2 mg/dL and Vit D deficiency (Vit D <25 nmol/L) or insufficiency (Vit D between 25 and 75 nmol/L). Exclusion criteria including; noncooperation of the patients during the study, Ca × Phosphorus >55mg^2^/dL^2^, of Vit D or Ca supplement consumption during 2 recent months and Ca>10 mg/dL. In 30 patients in case group, Vit D 50000 IU was weekly prescribed for 8 weeks. For 30 patients in control groups, placebo was prescribed similar to control group (weekly for 8 weeks). Demographic criteria of the patients were obtained and fasting blood sugar (FBS), glycosylated hemoglobin (HbA_1C_), Ca, phosphorus (P), Albumin, and 25 (OH) Vit D were checked in the beginning and at the end of the study in all of the patients. Urine protein was measured also in the end of the study for all of the patients.

### 3.2. Ethical issues

1) The research followed the tenets of the Declaration of Helsinki; 2) informed consent was obtained, and they were free to leave the study at any time and 3) the research was approved by the ethical committee of Shahrekord University of Medical Sciences (Ethical cod:1128 and IRCT code: IRCT2015081723656N1).

### 3.3. Statistical analysis

At the end of the study, data were entered to SPSS software, (version 21.0, SPSS Inc, Chicago, IL, USA). Then data were analyzed for comparisons of groups using the chi-square test, and independent and paired *t* test, and *P* values less than 0.5 were considered significant. All information was remaining confidential, so written consent forms were filled in by all cases. The study was done under permission and support of research deputy of Shahrekord University of Medical Sciences.

## 4. Results

Fifty-seven patients were participated in this study and 3 patients were left due to non-cooperation and withdrawal of one patient of case group and 2 patients of control group. Twelve patients of case group and 18 patients of control group were female and other were male (*P=*0.120). Mean age of case and control groups were 62.9±9.3 and 62.4±9 years respectively (*P=*0.85). At the beginning of study, mean serum level of Vit D in case and control groups were 36.76±19.16 (nmol/L) and 32.19±17.76 (nmol/L), respectively (*P=*0.33). However at the end of the study mean serum level of Vit D was 89.44±34.35 (nmol/L) and 38.02±23.90 (nmol/L) in the case and control groups, respectively (*P=*0.0001). There was a significant difference between serum level of Vit D before and after the study in the case group (*P=*0.001) as well as control group (*P=*0.02). As shown in Table 1, mean level of proteinuria in the patients of case group and control group were 962.62±885.99 mg/day and 755.71±640.94 mg/day, respectively (*P=*0.70). While, at the end of the study, it was 892.24±879.40 (mg/day) and 971.60±940.24, respectively (*P=*0.48). Difference of proteinuria before and after the study in the case and control groups were 70.38±553.71 and 215.89±451.44, respectively (*P=*0.028), which revealed a significant difference among them. There were no significant difference between two groups of the patients based on FBS, HbA_1C_, ESR and CRP levels (*P>*0.05).

**Table 1:** Comparison of variables in two groups of patients before and after the study

**Variable**		**Case group**	**Control group**	**P**
Vit D (nmol/L)	Before	36.76±19.16	32.19±17.76	0.326
	After	89.44±34.35	38.02±23.90	0.0001
	P	0.0001	0.02	-
	Before and after difference	52.68±27.48	5.82±14.08	<0.001
Proteinuria (mg/day)	Before	962.62±885.99	775.71±640.94	0.702
	After	892.24±879.40	971.60±940.24	0.482
	P	0.27	0.025	-
	Before and after difference	70.38±553.71	215.89±451.44	0.028
HbA_1c_ (%)	Before	7.82±0.86	8.14±1.26	0.318
	After	8.02±1.23	8.10±0.96	0.523
	P	0.259	0.895	-
	Before and after difference	0.2±0.99	0.04±1.05	0.406
FBS (mg/dL)	Before	146.69±53.03	151.50±52.35	0.571
	After	152.72±49.4	154.89±73.26	0.354
	P	0.482	0.882	-
	Before and after difference	6.03±35.54	3.39±60.05	0.544

## 5. Discussion

**Figure 1:**
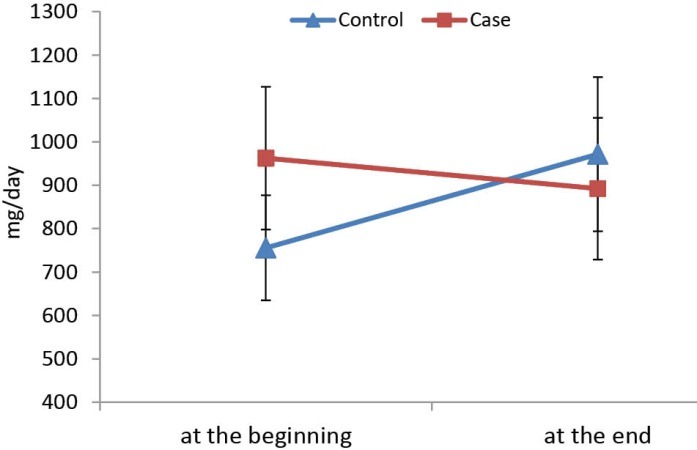
The mean of proteinuria during the study in the case and control group.

The study showed significant difference between changes of proteinuria between 2 groups of the patients during the study (*P=*0.028), thus we concluded that prescription of Vit D in type 2 diabetic patients with nephropathy and Vit D deficiency may decreased proteinuria (Figure 1).

Prevalence of Vit D deficiency is higher in diabetic patients, in addition association of Vit D deficiency or Vit D insufficiency with the DN was reported in some studies. (15,18). Vit D deficiency may be associated with other microvascular complication of diabetes such as diabetic retinopathy (19).

In addition to angiotensin converting enzyme inhibitors and angiotensin receptor blockers, other drugs such as spironolactone (aldosterone receptor blocker)(14), non- dihydropyridine calcium channel blockers (diltiazem), antihyperlipidemic agents (20), allopurinol (12) were used in the treatment of DN.

Effect of Vit D prescription in DN was evaluated in some clinical and experimental studies with controversial results. Ahmadi et al, in a clinical trial on 51 diabetic patients with DN and Vit D_3_ deficiency, it was found that Vit D_3_ prescription for three months had not any effect on decreasing of proteinuria (21).

Kim et al in the study on 63 diabetic patients with nephropathy and low level of serum Vit D during seven months showed that repletion with cholecalciferol could decrease albuminuria. They concluded that dietary Vit D in patients with DN may have a beneficial effect in delaying the progression of disease (22).

In a study by Bonakdaran et al, of 119 diabetic patients, 31 cases had Vit D deficiency, any significant reduction in the proteinuria after using of calcitriol for 8 weeks was detected (23).

In a study by De Zeeuw et al, on 281 diabetic patients, they could show that 2 mg/day of paricalcitol in addition of rennin-angiotensin-aldosterone blockers, could decrease proteinuria (24).

In another rstudy by Huang et al deficiency of serum 25(OH) Vit D_3_ levels was associated with microalbuminuria, and administration of cholecalciferol significantly decreased albuminuria in the early stages of treatment. They concluded that conventional doses of cholecalciferol may have antiproteinuric effects on Chinese diabetic patients (25).

We found no association between using of Vit D and serum Hb A_1c_, however different results were reported in other studies, for example, in Madar et al. 16-week administration of Vit D_3_ to healthy individuals with low Vit D status showed no improvement for HbA_1c_ (26). Likewise, Ahmadi et al reported no significant difference based on HbA_1C_ in both case and control groups (21). Otherwise Bonakdaran et al showed a significant reduction in HbA1c in diabetic patients (23).

## 6. Conclusions

Administration of Vit D in type 2 diabetic patients with Vit D deficiency or insufficiency leads to normalization of serum Vit D level and decrease proteinuria compared to control group, however we did not show any improvement of glycemic control indices in the patients. Thus, we concluded that, correction of Vit D deficiency may be an effective and safe modality of treatment for DN.

## Limitations of the study

Small proportion of the patients was a limitation of our study.

## Acknowledgments

This study is adapted from the internal medicine residency thesis of Mohsen Kabiri. Hereby, we thank the Research and Technology Deputy of Shahrekord University of Medical Sciences.

## Conflicts of interest

The authors declared no competing interests.

## Authors’ contribution

AM; study design, preparation of manuscript and final revision. MM; consultant of theses. MK; data gathering and data interpretation. SK; statistical consultant.

## Funding/Support

This study was a funded thesis at Shahrekord University of Medical Sciences (Grant# 1128).
